# The Information Needs of Adolescent Idiopathic Scoliosis Patients and Their Parents in the UK: An Online Survey

**DOI:** 10.3390/healthcare7020078

**Published:** 2019-06-20

**Authors:** Shaun Wellburn, Paul van Schaik, Josette Bettany-Saltikov

**Affiliations:** Centuria Building, Teesside University, Middlesbrough TS1 3BX, UK; J.B.Saltikov@tees.ac.uk (P.v.S.); P.Van-Schaik@tees.ac.uk (J.B.-S.)

**Keywords:** Scoliosis, adolescent, information, online, survey, patient, families, quality, individual, decision-making

## Abstract

Patients’ involvement in decision-making regarding their own health care is considered to be of great importance. However, their information needs are frequently reported to be unfulfilled. Few studies have investigated the knowledge, information and support needs of adolescent idiopathic scoliosis (AIS) patients and their families. Furthermore, previous studies have predominantly focussed on information needs relating to surgery. No previous studies have been conducted to specifically identify the information needs of AIS patients and their families. An online survey consisting of 18 questions was conducted to investigate the information needs of AIS patients and their families. Completed surveys of 83 participants (76 female, 7 male) from 44 differing postcode areas were analysed. The mean age of the respondents with scoliosis was 13.3 years (SD = 1.9; range = 10–18). Participants identified with feelings including worry, anxiety and being upset. The main information needs related to the cause and prognosis of the condition. Where participants had received information, there were contrasting views of the quality. The findings of this study stress the necessity for information materials to be accurate and applicable to each individual patient. Furthermore, the information should be presented in such a way as to be easily understandable, yet contain the necessary information required by AIS patients and their families.

## 1. Introduction

Patients’ involvement in decision-making and consultation have been on the political agenda in the UK for many years [1]. The Patients Charter [2], and later the National Health Executive [3], articulated the necessity for health-care professionals to actively involve patients in any decisions about their own health, as well as encouraging them to participate in their care. This notion of a shared decision-making paradigm is further supported in the literature [4,5,6,7]. For patients to be actively involved in decision-making, it is necessary for their information needs to be fulfilled. Previous research, however, suggests that patient information needs concerning treatment, across health conditions, remain unfulfilled [8,9,10]. It may be argued that to fulfill these information needs, it is first important to understand what they are. Further, that the best-placed people to understand the information requirements are the patients and their families themselves.

There is a dearth of studies in the field of scoliosis that have investigated the knowledge, information and support needs of adolescent idiopathic scoliosis (AIS) patients and their families. Previous studies that have been conducted in this field have predominantly focussed on patients’ [11,12,13] and their parents’ [14] experiences of a particular intervention, usually surgery. Two studies, those of MacCulloch et al. [15] and Khetani, Donaldson and Wright [16] have examined the information and knowledge needs of AIS patients. However, these studies focussed primarily on the information needs and knowledge relating to surgery. MacCulloch et al. [15] found that participants required information on a broad range of themes including recovery, post-surgical appearance and decision-making. Therefore, the results of these studies cannot be considered to represent the information and knowledge requirements of AIS patients at varying stages of progression of their condition.

A further study by van Schaik et al. [17] administered a postal questionnaire to idiopathic scoliosis patients (*n* = 83) and family members of persons with idiopathic scoliosis (*n* = 23) to examine their self-reported knowledge relating to 20 scoliosis-related topics. The 20 scoliosis-related topics covered a wide variety of areas including causes, types, diagnosis and progression of scoliosis, spinal surgery and bracing. Respondents were asked to rate, on a four-point Likert scale (poor–excellent), their level of self-reported knowledge of those topics. Results of this study showed that the level of self-reported knowledge was poor. However, this study was not limited to AIS patients but incorporated participants of all ages. The mean age of the persons with scoliosis responding to the questionnaire in the study of van Schaik et al. [17] was 41.9 years (SD = 17.5) with a range of 15 to 79 years. Therefore, the results of the study of van Schaik et al. [17] may not be truly reflective of the information needs of AIS patients.

To date, and to the authors’ knowledge, no studies have been conducted to specifically identify the information needs of AIS patients and their families. Therefore, this study aimed to identify the information needs of a national sample of AIS patients and their families through the application of a nationally available, online survey.

## 2. Methods

### 2.1. Participants

A total of 110 participants completed responses to the survey. This was a mixed-methods study incorporating open and closed questions. Twenty-seven of the completed surveys were not included in the final analysis as these related to persons with infant, juvenile or adult scoliosis and were therefore not applicable to the population of interest in this study. As a result, the survey responses of 83 participants were analysed in this study. Of these, 48 (58%) were completed by a person with scoliosis (PwS) themselves, and 35 (42%) were completed with the accompaniment of a parent or carer (PwSP). Seventy-six (92%) of the persons with scoliosis were female, and seven (8%) were male, the mean age of the respondents with scoliosis when first referred to the hospital was 13.3 years (SD = 1.9; range = 10–18). There was a broad range of durations since the person with scoliosis had their first referral to the hospital clinic (Range 0–6 months to more than 3 years).

### 2.2. Instrumentation

The authors of this study developed the questions for the online survey, and a selection of pre-determined responses to the questions, from the findings of a series of 15 semi-structured interviews, conducted as part of a larger body of work, exploring the information needs of AIS patients and their families [18]. The administered survey consisted of a total of 18 questions. The first eight questions dealt with non-personally identifiable, patient characteristics, such as age, sex, curve type and curve magnitude. The remaining questions focussed on each participant’s information needs and emotional responses. Where pre-determined responses were provided, with instruction to select one or all of those which applied, they were supported by free-text boxes for participants to enter alternative responses. A synopsis of the questions used in the survey is presented in Table 1.

### 2.3. Procedure

The final questions were inputted to Online Surveys (University of Bristol, Bristol, UK) so that the survey could be administered via the Internet. To attract respondents to the survey, after seeking and attaining consent, a link to the survey was posted on the Scoliosis Association UK Web site and also on the NHS Choices Web site for a period of six months. Implied consent was deemed to have been provided through participants choosing to complete the survey. Research ethics approval for the study was sought and obtained from the Teesside University Ethics Committee (031/12).

### 2.4. Analysis

Data were analysed for descriptive statistics and descriptive frequencies to quantify the results. Non-parametric Mann-–Whitney U tests were conducted to analyse any differences between responses completed by the person with scoliosis themselves and those completed with a parent. All analyses were conducted using SPSS version 22. A significance level of 0.05 was used for all statistical tests. Qualitative data were analysed with content analysis [19].

## 3. Results

Seventy-three participants provided valid data of their postcode area; from this data, a total of 44 differing postcode areas were identified indicating that the responses came from a broad geographical dispersion within the UK. To identify participants’ curve characteristics, a selection of options was provided, four for the curve type and five options for the curve size. These options and the corresponding results are shown in Table 2. The most common curve types were thoracic and lumbar; the most common ranges of curve size were 40° to 49° and 30° to 39°.

Respondents were asked to indicate, from a pre-determined list of seven options, all of the feelings which they had experienced when they were told they/their child had scoliosis. The pre-determined feelings and number of responses are shown in Table 3. Feelings of worry, anxiety and of being upset were the most frequently reported. Further responses identified from the free-text boxes included feelings of relief on receiving a diagnosis to explain the reason for the pain, sadness, frustration and also of guilt.

From a list of pre-determined options (Table 4) relating to specific information needs when they were told they/their child had scoliosis, participants were requested to indicate all those they deemed appropriate.

The most frequently identified information needs related to the *possible treatment options*, *what would happen now*, and *the effect of the condition on the child in later life*. For all pre-determined items in Table 4, there were a higher number of responses by the PwS themselves. None of the differences between the numbers of responses for the pre-determined items in Table 4 by the PwS and the PwSP proved to be statistically significant. Further information needs that were specified through the free-text box principally focussed on what effects the condition may have on the child’s current activities, particularly in relation to sport.

Sixty-five percent of respondents indicated that they had received information about their condition, though none specified whether they had received this information verbally, written or in an electronic format. Sixty-seven percent of participants who had received information rated that information as either satisfactory or very satisfactory. Ninety-one percent of those who did not receive any information indicated that they would have preferred to receive information about their condition. Furthermore, 77% of participants reported having searched the Internet for information relating to their (child’s) condition. More than 60% of the participants provided additional comments to the open questions, numbers 14 to 18.

Examples of responses given by participants who found the information satisfactory or very satisfactory included:

“There was very little information I needed after the consultation. In addition, I received a lot of detailed information about the actual operation, which I found useful.”

“Specialist nurse and consultant both gave detailed information and leaflets and contact information were also helpful. Before and after X-rays were shown and explained which was beneficial.”

However, comments made by participants who were dissatisfied with the information they received included:

“It (the information) was old and out of date.”

“Not enough info was given to us regarding a potential negative evolution of the situation and not all therapeutic options were illustrated. Confusing regarding need/role of gymnastics, physiotherapy. No explanation given to the child, this was left to parents to explain.”

Participants were asked to comment on how the information they received could be improved (Question 15). Many of the responses reiterated the necessity to answer the questions presented in Table 4. The overall feeling was that there was a general need for the provision of more information. The most common suggestion was the provision of written materials to take away from the consultation or from the general practitioner (GP) that may be referred to at a later date:

“Parents should be given a comprehensive information leaflet.”

“It would have been nice for more leaflets, booklets, etc.”

Furthermore, participants commented on how they would prefer to see the information prepared and presented:

“Some information maybe with pictures, something a bit friendlier, sort of tailored to people her age.”

An additional suggestion was that the information provided should be tailored to the patient and the stage of progression of their condition:

“It should be specific to the stage that you’re at, that would be important.”

Further comments related to raising the awareness of the condition in schools and emphasising that it was important to explain all of the treatment options available:

“Give detailed, accurate, comprehensive info on all treatment options and progression risks, including non-surgical treatment options.”

“We should have been given all of the options.”

## 4. Discussion

This study aimed to identify the information needs of a national sample of AIS patients and their families in the UK through the application of a nationally available, online survey.

The feelings experienced by participants in this study, presented in Table 3, were found to identify with similar feelings identified in other studies related to the diagnosis and treatment of scoliosis [14,17,20]. Feelings of sadness, frustration and guilt were also reported by respondents. The feeling of guilt principally related to not having noticed the problem earlier and could, most likely, be attributed to parents of the child. Similar to adolescents in the study by MacCulloch et al. [15], participants reported feelings of nervousness, although in the study of MacCulloch et al. [15] the participants had been prescribed surgery as their treatment. It could be argued that the feelings of nervousness in the current study may have resulted from either a fear of the unknown or a lack of knowledge following their diagnosis.

As with the pre-determined responses for feelings, the pre-determined responses for specific information needs proved to be relevant for the respondents to the online survey. All, except one (Is it hereditary?), of the information needs suggested were selected by over 50% of the respondents. Additionally, the pre-determined information need responses, developed from the face-to-face interviews, were reflective of scoliosis-related topics utilised in the study by van Schaik et al. [17]. Further, respondents in the study by van Schaik et al. [17] reported their knowledge of these topics as average or poor. It seems, therefore, unsurprising that these topics should be areas upon which participants in the current study required information.

A large proportion (65%) of respondents indicated that they had received information relating to scoliosis. Almost seventy percent of those who received information rated the information as either satisfactory or very satisfactory. This finding is in keeping with the results of a study by Sapountzi-Krepia et al. [21], who examined the experiences of patients undergoing brace treatment. This indicates that where information is provided, it would appear to be of relatively good quality. Nonetheless, others were dissatisfied with the information they were provided, deeming it to be old and out of date, not detailed enough, and confusing. These contrasting views on the quality of information may be indicative of a disparity of information, provided to AIS patients and their families, between different scoliosis clinics spread throughout the UK.

All of the respondents who had rated the information they had received as unsatisfactory, reported searching for further information on the Internet, although 80% of those who were satisfied with the information they received also searched the Internet for further information. This finding implies that there remained unfulfilled information needs or perhaps that patients and their parents required further reassurance that the information they received was accurate. The large number of participants that sought information from the Internet in the current study is reflective of the findings of Bull and Grogan [14] who reported that the Internet was the main source of information for their study participants. The overriding description, by the respondents in the current study of the information found in Internet searches, was that it was informative and helpful. Nonetheless, other respondents described the information they found as worrying, frightening, confusing and overall not very helpful. This finding suggests a lack of quality in terms of the available Internet information on scoliosis, a notion that is frequently reported in the literature [22,23,24], or a lack of information literacy [25,26].

Consequently, the question arises: How can the information provided by health care providers be improved? The predominant suggestions for inclusion of information provided, or made available, to AIS patients and their parents are what happens now? what treatment options are there for me? will I need an operation? will it get better? what is scoliosis? what causes scoliosis? and, is it hereditary? (see Table 4). The inclusion of information to fulfil these information needs would also improve the poor self-reported knowledge of the scoliosis related topics identified in the study by van Schaik et al. [17]. Respondents advocated the provision of written information materials, a finding also noted in work with cancer patients by Shea-Budgell et al. [27].

Further recommendations from participants in the current study included the necessity to provide scoliosis education in schools. This recommendation relates to suggestions for delivering education on posture and generally raising the awareness of the condition. Additionally, there was a recommendation for support resources for patients and parents. Suggestions for recommended support resources focussed around the provision of contact with others who have been through the same process and reports of success stories, a notion identified elsewhere in the literature [15]. Note that respondents made the recommendation for support through communication with others, even though only a small percentage of respondents indicated that they had sought support themselves. This may be because they were unable to identify an appropriate support resource. In addition, respondents suggested that information resources should be personalised and tailored to the specific needs of the patient.

The postcode details of the respondents indicated a broad geographical dispersion of the UK (44 differing postcode areas). This diversity of participant location increases the generalizability of the results to a wider population [28]. In addition, at the time of completing the survey, the length of time since the patients had first been referred to hospital varied widely between participants. This would suggest an extensive range of experience in terms of both diagnosis and subsequent treatment; from recently diagnosed patients to those who had potentially completed treatment.

Limitations of the study include the fact that recruitment was selective and restricted to visitors of the Scoliosis Association UK and National Health Service Choices Web sites, as these were the only places where the survey was posted. It could be argued, however, that these Websites were appropriate places to post this survey to capture the target participant group. A further limitation of the study was the lack of validation of the questions included in the survey. To compensate for this, further information was made available for some questions to enhance clarity and aid understanding. In addition, data regarding literacy, socioeconomic status and race/ethnicity were not collected. It is possible that these characteristics may have some bearing on the information needs of this population group. Despite these limitations, the strength of the work lies in the diversity of the postcode areas from which the respondents lived. Although it is recognised that a sample size of 83 respondents may be considered small in terms of a national survey, in terms of empirical work carried out in the field of scoliosis the sample is comparatively large.

## 5. Conclusions

The findings of this study provide a novel insight into the information needs of AIS patients and their families. The findings iterate the necessity for information materials to be accurate and applicable to the individual patient. Furthermore, the information should be presented in such a way as to be easily understandable yet contain the necessary information required by AIS patients and their families. As such, these findings may be used by clinicians to inform the way that they deliver information to this population group. Future work could explore any impact on these findings of the collection of additional data, such as literacy and socioeconomic status. In addition, preparing information materials based on these findings and with the addition of public and patient involvement.

## Figures and Tables

**Table 1:** Patient Survey Questions.

1. Who is completing this survey?
2. How old were you/your child when first referred to the hospital for scoliosis?
3. Is the person with scoliosis male/female?
4. Please enter the letters in the first half of your postcode.
5. Who first identified there may be a problem? (please do not enter their name)
6. How long is it since you/your child had the first referral appointment at the hospital?
7. What type of curve were you/your child diagnosed with?
8. How many degrees was the curve at the first referral?
9*. How would you describe your feelings on being told you/your child had scoliosis?
10*. Was a scoliosis specialist nurse present/available when you attended for your consultation?
11*. What were your specific information needs on being told that you/your child had scoliosis?
12. Did you receive any information about your (child’s) condition? 12a. If you did, was this information verbal, written, or electronic (websites)? 12b. Would you like to have received information about your condition?
13. If you received information, how would you rate it?
14*. Could you explain more about why you gave the information you received that rating?
15*. What in your opinion could be done to improve the information that you received?
16*. Did you search the Internet for information about your (child’s) condition?
17*. Did you seek emotional support?
18*. Do you have any suggestions about what would be important to include in information and leaflets to be given to scoliosis patients and their families?

* Indicates open question with free text box.

**Table 2:** Curve characteristics of participants.

**Classification**	***n***	**%**
Thoracic	28	34
Lumbar	23	27
Double-major	18	22
Thoracolumbar	14	17
**Curve Size**		
10°–19°	11	13
20°–29°	16	19
30°–39°	19	23
40°–49°	23	28
≥50°	14	17

**Table 3:** Distribution of responses to pre-determined feelings.

Feelings	PwS		PwSP		Total	
	*n*	%	*n*	%	*n*	%
Worried	31	23	18	20	49	21
Upset	26	19	16	17	42	19
Anxious	26	19	19	21	45	20
Nervous	17	12	9	10	26	11
Confused	15	11	11	12	26	11
Devastated	12	9	13	14	25	11
Annoyed	10	7	6	6	16	7

PwS—Person with scoliosis; PwSP—Person with scoliosis and parent/carer.

**Table 4:** Distribution of information needs responses.

Information Need	PwS		PwSP		Total	
	*n*	%	*n*	%	*n*	%
How will it affect me in later life?	36	15	26	14	62	14
What happens now?	34	14	28	16	62	14
What treatment options are there for me?	34	14	28	16	62	14
Will I need an operation?	33	14	22	12	55	13
Will it get better?	29	12	18	10	47	12
What is scoliosis?	28	12	19	11	47	12
What causes scoliosis?	25	10	22	12	47	12
Is it hereditary?	23	9	17	9	40	9

PwS—Person with scoliosis; PwSP—Person with scoliosis and parent/carer.

## References

[1] Le Var R.M.H. (2002). Patient involvement in education for enhanced quality of care. Int. Nurs. Rev..

[2] Department of Health (1992). The Patients Charter.

[3] National Health Service Executive (1999). Clinical Governance: Quality in the New NHS.

[4] Barry M., Edgman-Levitan P.A. (2012). Shared decision making-the pinnacle of patient-centred care. N. Engl. J. Med..

[5] Department of Health (2012). Liberating the NHS: No Decision about Me, without Me.

[6] Elwyn G., Frosch D., Thompson R., Joseph-Williams N., Lloyd A., Kinnersley P., Cording E., Tomson D., Dodd C., Rollnick S. (2012). Shared decision making: A model for clinical practice. J. Gen. Intern. Med..

[7] Stigglebout A.M., Van der Weijden T., De Wit M.P., Frosch D., Legare F., Montori V.M., Elwyn G. (2012). Shared decision making: Really putting the patients at the centre of healthcare. BMJ.

[8] Kidane B., Gandhi R., Sarro A., Valiante T.A., Harvey B.J., Rampersaud Y.R. (2011). Is referral to a spine surgeon a double-edged sword?. Can. Fam. Physician.

[9] Mordiffi S.Z., Tan S.P., Wong M.K. (2003). Information provided to surgical patients versus information needed. AORN J..

[10] O’Connor A.M., Drake E.R., Wells G.A., Tugwell P., Laupacis A., Elmslie T. (2003). A survey of the decision-making needs of Canadians faced with complex health decisions. Health. Expect..

[11] Rullander A.-C., Isberg S., Karling M., Jonsson H., Lindh V. (2013). Adolescents’ experience with scoliosis surgery: A qualitative study. Pain Manag. Nurs..

[12] Rullander A.-C., Jonssen H., Lundström M., Lindh V. (2013). Young people’s experiences with scoliosis surgery. A survey of pain, nausea, and global satisfaction. Orthop. Nurs..

[13] Koch K.D., Buchanan R., Birch J.G., Morton A.A., Gatchel R.J., Browne R.H. (2001). Adolescents undergoing surgery for idiopathic scoliosis: How physical and psychological characteristics relate to patient satisfaction with the cosmetic result. Spine.

[14] Bull J., Grogan S. (2010). Children having spinal surgery to correct scoliosis: A qualitative study of parents’ experiences. J. Health. Psychol..

[15] MacCulloch R., Donaldson S., Nicholas D., Nyhof-Young J., Hetherington R., Lupea D., Wright J.G. (2009). Towards an understanding of the information and support needs of surgical adolescent idiopathic scoliosis patients: A qualitative analysis. Scoliosis.

[16] Khetani N., Donaldson S., Wright J.G. (2008). What do patients and parents know about surgery for adolescent idiopathic scoliosis? A knowledge questionnaire. Spine.

[17] Van Schaik P., Flynn D., van Wersch A., Ryan K.-A. (2007). Factors important in the design of information material for scoliosis. Int. J. Rehabil. Res..

[18] Wellburn S. (2017). An Investigation of the Information Needs of Adolescent Idiopathic Scoliosis Patients and their Families. Ph.D. Thesis.

[19] Hsieh H.-F., Shannon S.E. (2005). Three Approaches to Qualitative Content Analysis. Qual Health Res..

[20] Salisbury M.H., LaMontagne L.L., Hepwoth J.T., Cohen F. (2007). Parents’ self-identified stressors and coping strategies during adolescents’ spinal surgery experiences. Clin. Nurs. Res..

[21] Sapountzi-Krepia D., Psychogiou M., Peterson D., Zafiri V., Iordanopoulou E., Michailidou F., Christodoulou A. (2006). The experience of brace treatment in children/adolescents with scoliosis. Scoliosis.

[22] Wellburn S., Bettany-Saltikov J., van Schaik P. (2013). An evaluation of Web sites recommended by UK NHS consultants to patients with adolescent idiopathic scoliosis at the first point of diagnosis. Spine.

[23] Nason G.J., Baker J.F., Byrne D.P., McCarthy T. (2012). Scoliosis-specific information on the Internet: Has the “Information Highway” led to better information provision. Spine.

[24] Mathur S., Shanti N., Brkaric M., Sood V., Kubeck J., Paulino C., Merola A.A. (2005). Surfing for scoliosis: The quality of information available on the internet. Spine.

[25] Gutierrez N., Kindratt T.B., Pagels P., Foster B., Gimpel N.E. (2014). Health literacy, health information seeking behaviors and Internet use among patients attending a private and public clinic in the same geographic area. J. Commun. Health..

[26] Bengeri M., Pluye P. (2003). Shortcomings of health information on the Internet. Health. Promot. Int..

[27] Shea-Budgell M.A., Kostaras X., Myhill K.P., Hagen N.A. (2014). Information needs and sources of information for patients during cancer follow-up. Curr. Oncol..

[28] Mathers N., Fox N., Hunn A. (2007). Surveys and Questionnaires. The NIHR RDS for the East Midlands/Yorkshire & the Humber.

